# Wilting may leave bees wanting: drops in turgor pressure may reduce viability of buzz-pollinated flowers

**DOI:** 10.1093/jxb/eraf061

**Published:** 2025-04-09

**Authors:** Benjamin S Lazarus, Agnes S Dellinger

**Affiliations:** University of Vienna, Department of Botany and Biodiversity Research, Rennweg 14, 1030 Vienna, Austria; University of Vienna, Department of Botany and Biodiversity Research, Rennweg 14, 1030 Vienna, Austria

**Keywords:** Biophysical modelling, buzz pollination, flower biomechanics, material properties

## Abstract

This article comments on:

**Alvord M, McNally J, Casey C, Jankauski M**. 2025. Turgor pressure affects transverse stiffness and resonant frequencies of buzz-pollinated poricidal anthers. Journal of Experimental Botany **76**, 1784–1794. https://doi.org/10.1093/jxb/erae504

This article comments on:

**Alvord M, McNally J, Casey C, Jankauski M**. 2025. Turgor pressure affects transverse stiffness and resonant frequencies of buzz-pollinated poricidal anthers. Journal of Experimental Botany **76**, 1784–1794. https://doi.org/10.1093/jxb/erae504

**Turgor pressure gives structural stability to plants as water in a cell pushes the cell membrane against the cell wall. This structural stability is particularly important in non-woody plant organs such as flowers, where high turgor pressure creates the hydrostatic skeleton required to maintain upright flowers. How changes in turgor pressure affect flower tissues and consequently their functioning during pollination have rarely been assessed. Leveraging techniques from biomechanics and modelling, Alvord *et al.* (2025) showcase a specialized pollination system, buzz pollination, and demonstrate that changes in turgor pressure critically change flower biomechanics and response to bee-like artificial vibrations.**

Buzz pollination is among the most enigmatic pollination systems in flowering plants, where mechanical vibrations in the range of 100–400 Hz are required to dislodge pollen from flowers (Vallejo-Marín, 2019). Approximately 50% of all bee species are capable of producing these vibrations, but not the honey bee *Apis mellifera* (Cardinal *et al.*, 2018). Buzz-pollinating bees apply these vibrations directly to the flowers by landing on the flowers and biting into the stamens while vibrating their thoracic muscles. The reason why pollen is only released through these specialized vibrations is that buzz-pollinated flowers conceal their pollen in tube-like structures such as poricidal anthers or anther cones (sometimes also corolla tubes). Thus, in contrast to longitudinally dehiscent anthers where pollen is displayed openly (common in ~90% of angiosperms), pollen in poricidal anthers (~10% of angiosperms) remains inside the anther structure until dislodged by the bee (Buchmann, 1983).

Despite increased interest in the functioning of buzz pollination in recent years, our understanding of the biomechanical aspects underlying buzz pollination remains fragmentary. This is largely due to the fact that both bee traits and floral traits seem to play an important role in modulating pollen release (how much pollen is removed from an anther in a given interaction) and both bee and floral traits have a number of parameters that can vary. For example, the vibrations produced by bees are often characterized by three properties, the duration of the buzzing, the maximum vibration amplitude (measured in displacement, velocity, or acceleration), and the frequency (how many vibration cycles occur per second). Each of these vibration properties can vary between bee species and individuals (Rosi-Denadai *et al*., 2020), and bees may even adjust their vibration properties (i.e. duration of buzz) during flower visits (Buchmann and Cane, 1989). Bees further differ in their behaviour on the flowers, with some bees vibrating entire flowers, single stamens, or just stamen tips (Mesquita-Neto *et al*., 2018). All vibration properties as well as bee behaviour (vibration location, grip strength) are likely to have have strong effects on pollen release. Longer buzz duration and higher amplitude, for example, seem to release more pollen, while the effect of frequency is still debated. Studies have suggested that buzzing at the proper frequency could achieve resonance in vibrated anthers, particularly when bee mass is considered (Jankauski *et al.*, 2022). Resonance occurs when consecutive vibrations constructively interfere with each other, leading to significant amplifications of the amplitude. The frequency at which this occurs is dependent on the anther morphology and flower material properties, which can vary significantly among plant species.

This leads into the other side of the plant–pollinator interaction: the flower. Like bee vibrations, floral traits exhibit a number of properties that can control their response to buzzing (Arroyo-Correa *et al*., 2019). The study by Alvord *et al.* (2025) focuses on the most common and best-understood buzz-pollinated floral morphology, flowers from the genus *Solanum*, in which corollas are reflexed, and stamens are usually aggregated in the centre of the flower (often forming a cone) and have smooth anther walls (Vallejo-Marín *et al.*, 2022). Experimental studies have shown that anther cones (present in many *Solanum* species, but not the focal species of Alvord *et al*., 2025) ideally transmit the bee’s mechanical vibrations and lead to high rates of pollen release (Kemp and Vallejo-Marín, 2021). While such solanoid flowers have evolved repeatedly across angiosperms, many other morphologies exist among buzz-pollinated flowers, with variable corolla shapes, more complex, loose stamen arrangements, variable anther orientations, and differently structured anther walls (smooth, corrugated, stiff, soft, etc.) and diverse anther appendages (Fig. 1; Kopper *et al*., 2024).

**Fig. 1.**
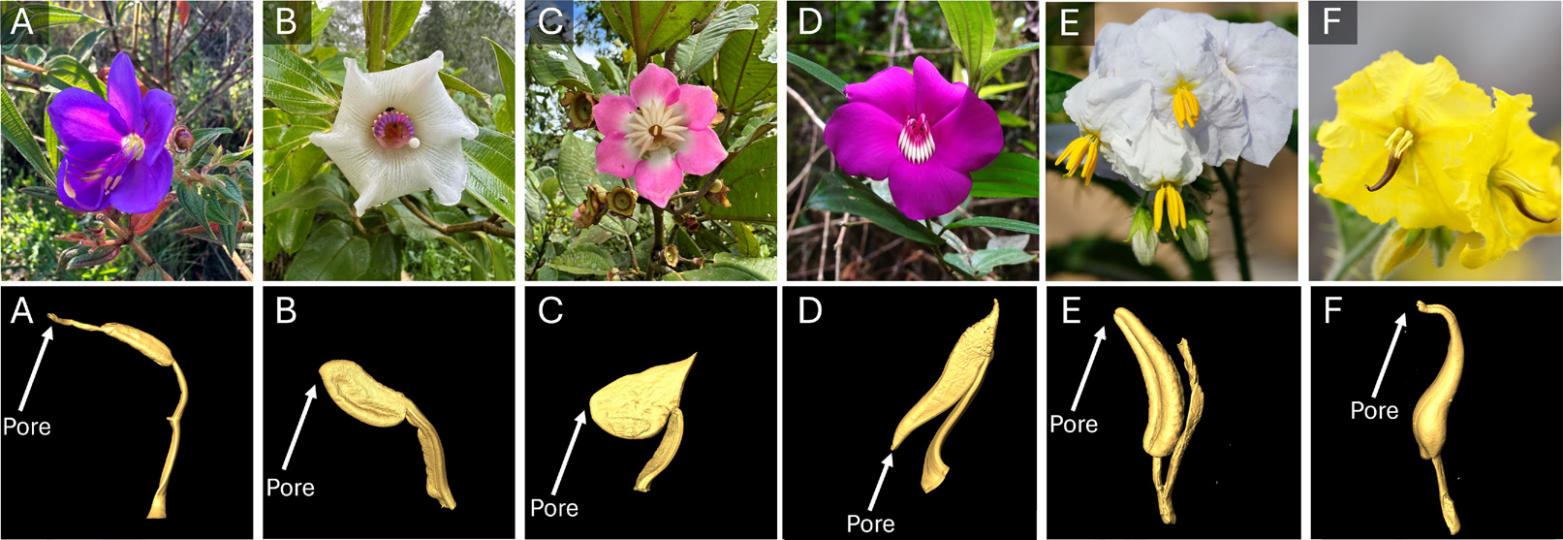
Buzz-pollinated flowers exhibit a wide range of diversities. Buzz-pollinated flowers and their stamens come in a tremendous diversity of shapes and sizes. The role of these disparate morphologies is still poorly understood, with hypotheses ranging from pollination niche partitioning (i.e. specialization on different buzzing bees) to pollen dosing (limiting pollen release) to differential pollen placement on bees and beyond. This figure provides a few examples of the diversity seen in buzz-pollinated flowers of two of the largest radiations of buzz-pollinated flowers, the *Melastomataceae* plant family (>5800 spp.) and the genus *Solanum* (>1200 spp.), with corresponding CT scans of their stamens shown in the lower panels. The species shown, from left to right, are (A) *Rhynchanthera grandiflora* (Aubl.) DC., (B) *Blakea involvens* Markgr., (C) *Blakea* sp. nov*.*, (D) *Meriania speciosa* (Bonpl.) Naudin, (E) *Solanum sisymbriifolium* Lam. (study species of Alvord *et al*., 2025, photo credit Mario Vallejo-Marín), and (F) *Solanum rostratum* Dunal (photo credit Mario Vallejo-Marín). Other notable morphologies not shown in this figure include *Senna* which utilize curved petals to ricochet pollen onto buzzing visitors and *Pedicularis* species which have tubular petals, rather than anthers, which restrict pollen release to a narrow pore that must be buzzed to extract pollen.

With such a broad range of floral traits on the one side, and bee buzzing parameters (i.e. amplitude, frequency, duration, gripping location, and bee mass) on the other side, researchers have struggled to disentangle which aspects of the buzzing interaction modulate stamen movement and, consequently, pollen movement (in the anther) and hence pollen release. The recent study by Alvord *et al.* (2025) provides the missing link between the bee side and the floral side of the interaction by connecting applied vibrations to the mechanical behaviour of the anther through a biophysical model. Biophysical models provide a tantalizing approach to understanding such complex interactions by allowing researchers to simplify the system and isolate the effects of individual floral or buzzing traits (Jankauski *et al.*, 2022). However, these models are limited by available experimental data to use as inputs. To date, most simulations utilize idealized billiard models where particles bounce around in a vibrating 2D box (representing a simplified locule) until they escape via a narrow pore (Buchmann and Hurley, 1978; Hansen *et al.*, 2021; Borin *et al.*, 2024). While this provides some information on how certain stamen traits (i.e. pore size) control pollen release, it provides little insight into how bee interactions affect the stamen and therefore pollen movement. The models developed by Alvord *et al*. (2025) push the field forward by allowing researchers to explore more nuanced variations between species such as how differences in porosity, material properties, or second moment of area might combine to modulate anther movement, while previous models primarily accounted for the effects that topological differences (i.e. anther length and pore width) may have on pollen escape in a moving anther. A prime opportunity exists to combine the Alvord *et al*. (2025) model for anther motion with existing models for pollen escape. This will create a continuous connection from a bee vibration to the resulting anther motion and, ultimately, to pollen release.

In order to establish realistic biophysical models of anther motion during buzz pollination, two main factors on the plant side of the interaction need to be quantified: the architecture of the anther, namely porosity and second moment of area (essentially the cross-sectional ‘shape effect’ resistance to bending), and the material properties of the tissue— density and Young’s modulus (which can be thought of as the tissue stiffness). Alvord *et al.* (2025) developed models that integrate both of these. On the morphological side, the authors simplify the anther structure to a circular cross-section with four cylindrical cavities, representing the locules, travelling the length of the anther. The authors account for tissue porosity by introducing a variable, β, that represents the ratio of solid to porous area in the anther wall. This idealized shape and porosity are used to calculate an effective cross-sectional area and effective second moment of area, which represents only the solid material, for their model. To ascertain material properties, Alvord *et al.* (2025) performed their own measurements. Density can be straightforward to measure (and the authors find a density value for fresh anthers that is reasonably only slightly above that of water), but to measure the stamen’s transverse stiffness, the authors fabricated a custom fixture to perform flexural tests on the small and soft stamens. Porosity and turgor pressure were not experimentally measured.

Alvord *et al*. (2025) used these measurements and assumptions to develop a static model that describes how an anther would deform under a stationary load (such as a bee’s mass). The authors then extended the static model beyond the quasi-static regime to include dynamic effects (differences in movement when the material is deformed quickly) and to better understand vibratory properties, such as anther resonant frequencies. The authors leveraged a technique called dynamic nanoindentation to measure how the stiffness of the anther changes as it is vibrated more rapidly. The results of this test were then paired with experimental modal analysis, where the anther’s resonant frequencies are measured using a shaker setup (artificially vibrating the anther). The resonant frequencies and transverse stiffness are used as validation to determine the range of possible porosity ratios and turgor pressures found in the anther. The resulting dynamic model can predict the resonant frequency of a vibrating anther which could have implications for pollen release (Jankauski *et al.*, 2022). Further, this model predicts how the resonant frequency might change should other parameters such as anther traits vary (i.e. among species: porosity, material properties, or cross-section) or change (i.e. changes in turgor pressure due to changes in the environment). Meanwhile, the static model predicts how the transverse stiffness might change should any of these variables vary, which will affect the achievable amplitudes and ultimate pollen release that occur during buzz pollination.

The model by Alvord *et al*. (2025) allows for exploring effects of morphological variation [i.e. among-species variation in porosity, material properties (such as stiffness or density), and cross-section] as well as of changes in floral traits (i.e. environment-induced changes in turgor pressure and viscoelasticity) on pollen release. While the current dynamic model does not include a driving force (i.e. a bee vibration), it can be used as a blueprint for predicting the displacement of anthers of different morphologies under different vibration conditions. For example, future studies could follow the same approach as Alvord *et al*. (2025) to create a (simplified) geometric model of different buzz-pollinated stamen morphologies and could add a driving force term that would allow for the application of different vibration regimes (i.e. varying frequencies and amplitudes). Such models could also be used to explore how changing bee vibrations (such as smaller amplitudes or different frequencies) would affect the movement of stamens of different morphologies. For example, do changes in bee buzzing amplitude affect maximum displacement (a parameter likely to be linked to increased pollen expulsion) more strongly in some stamen morphologies than others? These models can even be extended to explore how changes in a stamen’s biomechanical properties (i.e. decrease in stiffness or density) might change the stamen’s response to a vibration. Finally, these models may be used to approximate stamen traits (such as stiffness or second moment of area) from vibration response data.

The other set of floral traits assessed by Alvord *et al*. (2025) in their models are biomechanical floral properties which are highly affected by external environmental conditions, such as turgor pressure (the force with which the plasma membrane is pushed against the cell wall) and viscoelasticity (property of material that exhibits both viscous and elastic characters). Turgor pressure is maintained by the osmotic flow of water in the plant and is sensitive to changes in water regimes (i.e. drought leading to tissue desiccation, Beauzamy *et al*., 2014). In non-woody plant organs such as flowers, in particular, where turgor pressure gives structural stability to the tissue, changes in turgor pressure have immediate effects on the material properties of the flower, with possible changes in floral shape or vibrational properties. Accordingly, Alvord *et al.* (2025) demonstrate that reductions in turgor pressure change the stamen’s resonant frequency, with unknown effects on pollen release. Besides the study of Alvord *et al.* (2025), however, turgor pressure has received little (experimental) attention by pollination biologists. On the simplest level, turgor pressure can modulate the stiffness of a material (Kanahama *et al.*, 2023), with higher pressure increasing the stiffness of the structure and requiring more force to bend it. The potential effects in the context of buzz pollination are unclear, where both larger vibration amplitudes (Vallejo-Marín, 2019) and better vibration transmission (Woodrow *et al.,* 2024) have been shown to increase pollen release. On the one hand, a stiffer anther may limit the vibration amplitudes a certain bee with a fixed maximum force output can achieve on a stamen. However, a stiffer stamen may also improve the transmission of vibrations and potentially result in higher rates of pollen release.

Turgor pressure is directly linked to viscoelasticity, which may also greatly affect vibration transmission during buzz pollination. A common and useful assumption in mechanics is to envisage materials as springs which have a linear relationship between the force applied and the resulting movement. Viscoelastic fluid-filled structures (such as plant tissues) do not obey this assumption. The fluid within the material can flow and change the local properties as the material is deformed. However, this process does not happen instantaneously. The result is that biological materials have a time-dependent response to mechanical forces; that is, if a force is applied slowly, the material will have time to shift and relax, which will lead to a different motion response from that if the force is applied quickly. In the case of a buzz-pollinated anther, this causes the anther to stiffen when vibrated at higher frequencies. Functionally, this could modulate the amplitude of vibrations experienced by an anther and the resulting pollen expulsion. Alvord *et al.* (2025) explore this with their measurements of the dynamic and static moduli and their corresponding models. Future tests could expand on their results, exploring the frequency dependence of viscoelastic energy dissipation during vibration, and how viscoelasticity changes during a flower’s life span.

## Perspective: buzz pollination and climate change—wilted flowers as the norm?

**Fig. 2.**
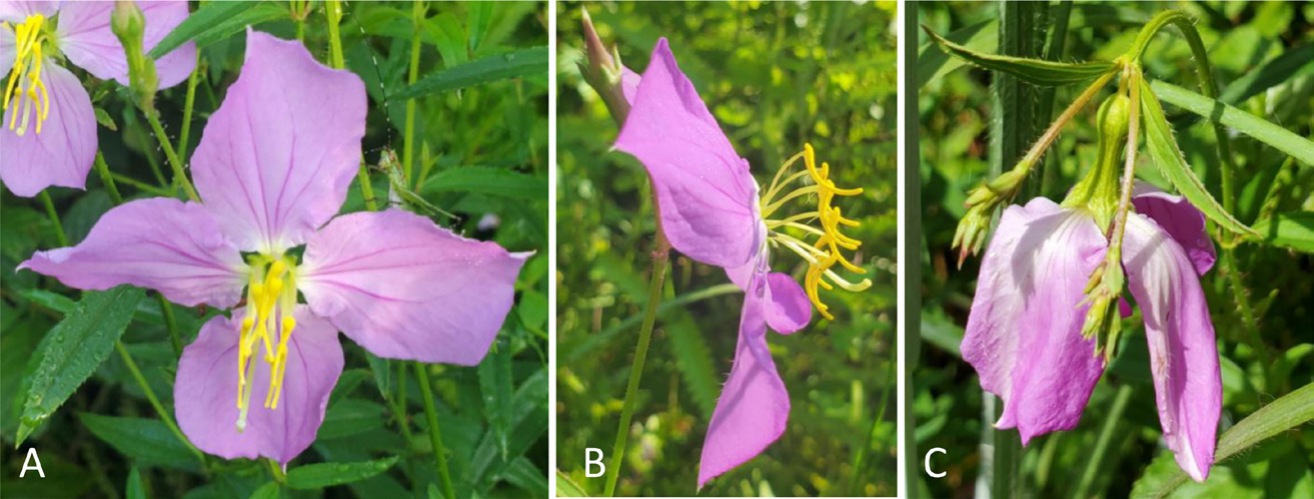
Turgor loss in flowers is a common phenomenon on hot days: *Rhexia nashii* Small (Melastomataceae). *Rhexia* flowers open shortly before sunrise (~05.30 h in July in NE Florida, USA) when it is still relatively cool (~20–25 °C). They are visited and buzzed immediately by buzz-pollinating bumble-bees (especially *Bombus impatiens* Cresson, 1863 and *Bombus pensylvanicus* (De Geer, 1773), and, a little later during the day, carpenter bees (*Xylocopa* sp.). In the freshly opened flowers, petals are spreading and stamens are stiff and arranged around the centre of the flower (A, B). After only a few hours of sun exposure and increasing heat (~10 00 h, >30 °C), flowers lose turgor pressure and start wilting, leading to bent-over flowers with pseudo-campanulate petals (C). Visitation rates by bumble-bees drop markedly at that time, but some individuals continue visiting and buzzing wilted flowers (see Video 1). Our preliminary investigations have shown that such flowers barely release pollen, probably due to the loss in turgor pressure and altered biomechanical properties. Handling time per flower further seems to increase for bees since they have more trouble landing on the pendant flowers. Overall, how extreme temperatures change bee foraging behaviour and whether hot days starve bees by temporally and biomechanically restricting their foraging remains to be tested.

The finding by Alvord *et al.* (2025) that turgor pressure reduction drastically changes a stamen’s resonant frequency and potentially pollen release dynamics has serious implications for plant–pollinator interactions in light of climate change. This effect might be particularly acute in buzz-pollinated flowers given the finding that higher turgor pressure provides an additional stiffening effect at higher oscillatory frequencies. Furthermore, buzz-pollinated plants have recently been described as particularly dominant in arid regions (Russell *et al.*, 2024), prominently exposing them to climate change-related disruptions in precipitation regimes and heatwaves. From our own research, we know that flowers of the buzz-pollinated genus *Rhexia* (*Melastomataceae*) lose floral turgor pressure within only a few hours of sun and heat exposure, changing floral displays from open, upright flowers to pseudo-campanulate pendant flowers (Fig. 2). This change occurs while bees are foraging, and bees keep visiting wilted flowers. Yet, in experimental assessments when applying artificial vibrations to wilted flowers, very little pollen is released relative to fresh, hydrated flowers. We have also noticed that during periods of uncharacteristic heat, pollen release seems to decrease significantly, suggesting that buzz pollination may be a particularly vulnerable pollination system in the context of climate change. Given that ~8% of flowering plants, including several important agricultural crops (i.e. tomatoes, eggplants, kiwi fruits, and blueberries; Cooley and Vallejo-Marín, 2021), are buzz pollinated, environment-induced changes in flower biomechanics warrant immediate attention. With their dynamic model, Alvord *et al.* (2025) provide a critical tool for exploring how changes in environmental conditions affect flower biomechanics and pollen release across the large diversity of buzz-pollinated flowers.
